# Proteomic Analysis of Drug-Resistant Mycobacteria: Co-Evolution of Copper and INH Resistance

**DOI:** 10.1371/journal.pone.0127788

**Published:** 2015-06-02

**Authors:** Yuling Chen, Fan Yang, Zhongyuan Sun, Qingtao Wang, Kaixia Mi, Haiteng Deng

**Affiliations:** 1 MOE Key Laboratory of Bioinformatics, School of Life Sciences, Tsinghua University, Beijing, China; 2 Beijing Chaoyang Hospital Affiliated to Capital Medical University, Beijing, China; 3 CAS Key Laboratory of Pathogenic Microbiology and Immunology, Institute of Microbiology, CAS, Beijing, 100101, China; Murdoch University, AUSTRALIA

## Abstract

Tuberculosis, caused by the pathogen *Mycobacterium tuberculosis*, is a worldwide public health threat. *Mycobacterium tuberculosis* is capable of resisting various stresses in host cells, including high levels of ROS and copper ions. To better understand the resistance mechanisms of mycobacteria to copper, we generated a copper-resistant strain of *Mycobacterium smegmatis*, mc^2^155-Cu from the selection of copper sulfate treated-bacteria. The mc^2^155-Cu strain has a 5-fold higher resistance to copper sulfate and a 2-fold higher resistance to isoniazid (INH) than its parental strain mc^2^155, respectively. Quantitative proteomics was carried out to find differentially expressed proteins between mc^2^155 and mc^2^155-Cu. Among 345 differentially expressed proteins, copper-translocating P-type ATPase was up-regulated, while all other ABC transporters were down-regulated in mc^2^155-Cu, suggesting copper-translocating P-type ATPase plays a crucial role in copper resistance. Results also indicated that the down-regulation of metabolic enzymes and decreases in cellular NAD, FAD, mycothiol, and glutamine levels in mc^2^155-Cu were responsible for its slowing growth rate as compared to mc^2^155. Down-regulation of KatG2 expression in both protein and mRNA levels indicates the co-evolution of copper and INH resistance in copper resistance bacteria, and provides new evidence to understanding of the molecular mechanisms of survival of *mycobacteria* under stress conditions.

## Introduction

The ancient pathogen *Mycobacterium tuberculosis* causes tuberculosis (TB) in one third of the world’s population and is one of the formidable threats to human health [1]. *M*. *tuberculosis* is capable of resisting survival stresses and stays alive in host cells for years that causes the prevalence of TB. The emergence and widespread of multidrug-resistant and extensively drug-resistant TB has also raised great concerns for public health [2–4]. The IFN-γ–mediated activation of macrophages is the major immune response of the host cells to *M*. *tuberculosis* infection, in which *M*. *tuberculosis* residing in phagosomes was cleared by a range of hydrolytic enzymes, bactericidal peptides, and reactive oxygen and nitrogen intermediates in phagolysosome [5–6]. Recent studies have demonstrated that copper ions in host cells possess bactericidal effects against invading mycobacteria [7–8].

It has been broadly accepted that copper ions are an essential nutrient required for survival by all organisms from bacteria to humans. Copper ions are a co-factor of enzymes that undergo reversible oxidation from Cu(I) to Cu(II) in electron transfer reactions. Both Cu/Zn-superoxide dismutase SodC and the cytochrome c oxidase in *M*. *tuberculosis* use copper as a cofactor [9–10]. However, excess copper ions are detrimental to the survival of bacteria by interacting with hydrogen peroxide to form hydroxyl radicals and by binding to proteins [11]. Thus, host cells are capable of eliminating intracellular bacteria by taking the advantages of toxic properties of copper ions. For example, IFN-γ–activated macrophages traffic the Cu transporter ATP7A to vesicles that fuses with phagosomes to increase Cu content and the bactericidal activity against Escherichia coli [12]. Infection of macrophages with *M*. *avium* increases the copper ion concentrations within the phagosomal compartment to control growth of mycobacteria [13]. Accumulation of copper ions was observed in the granulomatous lesions of infected lungs in a guinea pig model of infection and more importantly, copper resistance was found to be essential for virulence of *M*. *tuberculosis* [14].

*M*. *tuberculosis* is an intracellular pathogen that has evolved many strategies to evade host’s immune surveillance. For example, phagosomes containing *M*. *tuberculosis* have limited fusion with the lysosome in macrophages [15–16]. *M*. *tuberculosis* also has acquired independent mechanisms to protect its survival from the copper ion-induced toxicity [14, 17]. Studies have shown that metal cation transporter p-type ATPase (CtpV) is a copper responsive gene that encodes a Cu efflux protein located in the inner membrane of mycobacteria and is required for virulence of *M*. *tuberculosis* [18–19]. A recent study shows that the copper transporter (MctB) is a pore-forming protein located in the outer membrane (OM) of *M*. *tuberculosis* [14] and prevents the accumulation of Cu within the mycobacterial cell probably by efflux of cuprous ions. Using native mass spectrometry, Gold et. al. showed that mycobacterial metallothionein MymT was an important component of Cu homeostasis in *M*. *tuberculosis* by binding to copper ions [20]. The Cu repressor (RicR) regulon and its targeted genes are required simultaneously to combat Cu toxicity in vivo [17, 21].

A few studies have analyzed the global responses of mycobacteria to copper ion-induced toxicity. DNA microarrays were used to profile mycobacterial responses to copper ions and identified 30 copper-responsive genes in *M*. *tuberculosis* [22]. Characterization of the copper-induced changes in mycobacterial proteomes is also important to understanding mechanisms of *M*. *tuberculosis* survival under stress and to provide new targets for controlling this virulent pathogen. In this study, we setup a screening method for selection of the copper-resistant strain from *M*. *smegmatis* strain mc^2^155, and obtained a resistant strain, named mc^2^155-Cu, which hasa 5-fold higher resistance to copper ions than mc^2^155. Our result provided evidences that copper-resistant strain has a slow growth trait and is also resistant to isoniazid (INH), as compared to mc^2^155. On the basis of quantitative proteomic analysis, we showed that the increased copper and INH resistance in mc^2^155-Cu was resulted from the up-regulation of copper-translocating P-type ATPase expression and the down-regulation of KatG2 expression.

## Materials and Methods

### Chemicals and reagents

7H9 liquid medium powder, isoniazid, deoxycholate and iodoacetamide (IAA) were purchased from Sigma (St Louis, MO). Middle Brook 7H10 Agar was purchased from Hopebio (Qingdao, China). Albumin, D-glucose, dithiothreitol (DTT), BCA protein assay kit were purchased from Solarbio (Beijing, China). Glycerol and CuSO_4_ was from Xilong (Shantou, China). Sequencing grade trypsin was purchase from Promega (Fitchburg, WI). The Total RNA Isolation System and Reverse Transcription kits were purchased from TIANGEN (Beijing, China). SYBR mixture was purchased from Genstar (Beijing, China).

### Bacterial strains and growth conditions

*M*. *smegmatis* strains were grown in Middlebrook 7H9 liquid medium supplemented with 10% ADS (50 g/L albumin, 20 g/L D-glucose and 8.1 g/L sodium chloride), 0.05% Tween 80 and 0.2% glycerol or on Middlebrook 7H10 agar supplemented with 0.5% glycerol and 10% ADS. CuSO_4_ and isoniazid were added to the concentrations needed.

### Selection of copper-resistant *M*. *smegmatis* strain

Bacteria were diluted 1:100 into 5 ml 7H9 with 10% ADS and 50 μM CuSO_4_ added. The cultures were grown to log phase and diluted 1:100 into 5 ml 7H9 containing 60 μM CuSO_4_. This process was repeated until the concentration of CuSO_4_ reached 300μM. In order to obtain a stable copper-resistant bacterial strain, the bacteria were sub-cultured for 10 generations and spread on 7H10 plates containing 300 μMCuSO_4_. Single colony was then incubated in liquid media and the minimal inhibition concentration (MIC) of copper was measured.

### Measurement of bacterial growth

To measure the growth curve of M. smegmatis mc^2^155 and copper resistant strain, bacteria mc^2^155-Cu were grown to OD_600_ of 0.2–0.5, and diluted 1:1000 in media without CuSO_4_. The OD_600_ values of cultures were measured with a spectrophotometer every 3 hours. All the measurements were replicated three times.

### Determination of MICs to copper and isoniazid

The measurement of the minimum inhibitory concentrations (MICs) was performed on solid media plates. 10 μL of a 10^5^-cells/ml suspension of *M*. *smegmatis* strains was streaked on 7H10 plates with different concentrations of CuSO_4_ or isoniazid. Plates were incubated at 37°C for 4 days. Then the numbers of viable bacteria were counted. The MIC was defined as the lowest concentration of drug that inhibited the visible bacterial growth of *M*. *smegmatis* after 4 day incubation. All assays were repeated three times.

### Isoniazid resistant assay

Bacteria were added with 0.1 mg/ml isoniazid for 3 h, and diluted several times. 20 μL of a 10^5^-cells/ml bacteria were spread on 7H10 plates, and incubated at 37°C for 4 days. The number of viable bacteria was counted to determine the survival rate of bacteria. A two-sided t-test was used to determine whether the copper-resistant strain was also resistant to isoniazid as compared to mc^2^155 strain.

### Metabolomic analysis

Bacteria were collected and washed with ice-cold PBS twice. Then bacteria were metabolically quenched in precooled H_2_O/ACN/methanol (2:4:4) and lysed by grinding in Beadbeater for 3 min. Cellular debris was removed by centrifugation at 12000 rpm for 30 min. The concentrations of bacterial metabolites were estimated by the protein content determined with the BCA assay. One part of samples was labeled with the TMT reagents to quantify the amine-containing molecules, and the other part was dried and analyzed by LC-MS/MS. For TMT labeling, the samples were dried and redissolved in 50 μL 200 mM tetraethylammonium bromide (TEAB), and incubated with TMT reagent for 1 h at room temperature and the reaction was quenched with 5% hydroxylamine. The samples were stored at -80° for LC-MS/MS analysis. For LC-MS/MS analysis of metabolites, samples were separated by RP chromatography and analyzed in both negative mode and positive mode using a Dionex U3000 HPLC coupled to a Q Exactive mass spectrometer. The data were collected using the Xcalibur 2.1.3 software in data-dependent acquisition mode.

### Proteomic Analysis

Bacteria were washed twice with PBS, and lysed with 8 M Urea in PBS. Protein concentrations were measured by the BCA method. Equal amount of proteins from *M*. *smegmatis* mc^2^155 and copper-resistant *M*. *smegmatis* (100 μg) were reduced with10 mM dithiothreitol (DTT) and alkylated with 25 mM iodoacetalmide (IAM). Samples were diluted with PBS to 1.5 M Urea followed by digestion with trypsin of a 1:100 protease/protein ratio at 37°C overnight. The samples were desalted by Oasis HLB columns (Waters, MA). Peptides from different samples were labeled with tandem mass tags (TMT) reagents (Thermo, Pierce Biotechnology) according to the manufacturer’s instruction. Briefly, the TMT reagents were dissolved in acetonitrile and added to the peptide solution. The reaction was kept at room temperature for 1 hour, and quenched by 5% hydroxylamine for 15 min. The TMT labeled peptides were mixed and desalted by HLB column.

The peptides were fractionated by a UPLC3000 system (Dionex, CA) with a XBridgeTM BEH300 C18 column (Waters, MA). Mobile phase A is H_2_O with ammonium hydroxide, pH 10; and mobile phase B is acetonitrile in ammonium hydroxide pH 10. Peptides were separated with the followed gradients: 8% to 18% phase B, 30 min; 18% to 32% phase B, 22 min. 48 fractions were collected, dried by a speedvac, combined into 12 fractions, and redissolved in 0.1% formic acid.

For quantitative proteomic analysis, the TMT-labeled peptides were separated by a 60-min gradient elution at a flow rate of 0.250 μl/min with an EASY-nLCII integrated nano-HPLC system (Proxeon, Denmark), which is directly interfaced with a Q Exactive mass spectrometer. The analytical column was a fused silica capillary column (75 μm ID, 150 mm length; packed with C-18 resin, Lexington, MA). Mobile phase A consisted of 0.1% formic acid and mobile phase B consisted of 100% acetonitrile and 0.1% formic acid.

The Q Exactive mass spectrometer was operated in the data-dependent acquisition mode using the Xcalibur 2.1.3 software and there was a single full-scan mass spectrum in the Orbitrap (300–1800 m/z, 70 000 resolution) with automatic gain control (AGC) target value of 3e6. A data-dependent acquisition method was performed to collect generated MS/MS spectra at 17500 resolution with AGC target of 1e5 and maximum injection time (IT) of 60 ms for top 10 ions observed in each mass spectrum. The isolation window was set at 2 Da width, the dynamic exclusion time was 60 s and the normalized collisional energy (NCE) was set at 30.

### Data analysis

The generated MS/MS spectra were searched against the *M*. *smegmatis* mc^2^155 database that has 6578 entries from Uniprot (http://www.uniprot.org/uniprot/?query=taxonomy:246196) using the SEQUEST searching engine of Proteome Discoverer software (version 1.4). The search criteria were as follows: full tryptic specificity was required; one missed cleavage was allowed; carbamidomethylation (C) and TMT sixplex (K and N-terminal) were set as the fixed modifications; the oxidation (M) was set as the variable modification; precursor ion mass tolerances were set at 10 ppm for all MS acquired in an orbitrap mass analyzer; and the fragment ion mass tolerance was set at 20 mmu for all MS2 spectra acquired. The peptide false discovery rate was calculated using Percolator provided by PD. When the q value was smaller than 1%, the peptide spectrum match was considered to be correct. False discovery was determined based on peptide spectrum match when searched against the reverse, decoy database. Peptides only assigned to a given protein group were considered as unique. The false discovery rate was also set to 0.01 for protein identifications. Relative protein quantification was performed using Proteome Discoverer software (Version 1.4) according to manufacturer’s instructions on the six reporter ion intensities per peptide. Quantitation was carried out only for proteins with two or more unique peptide matches. Protein ratios were calculated as the median of all peptide hits belonging to a protein. Quantitative precision was expressed as protein ratio variability. Differentially expressed proteins were further confirmed by qPCR. Proteomics data have been deposited to the ProteomeXchange Consortium via the PRIDE partner repository with the dataset identifier PXD001989.

### Quantitative Real-Time PCR (qPCR)

The mc^2^155 and mc^2^155-Cu strains were cultured in 7H9 medium and collected when OD_600_ reached 1.0. Total RNA was extracted using RNAprep pure Cell / Bacteria Kit. cDNA was synthesized from 3μg total RNA with the Reverse transcription kit. Quantitative real-time PCR was performed with the Roche LightCycler 480II Detection System using SYBR green SuperRealPremixs. RNA polymerase sigma factor rpoD was used as an internal control. Relative expression levels for each reference gene were calculated. The relative expression ratio of a target gene was calculated based on the threshold cycle (Ct) deviation of mc^2^155-Cu versus mc^2^155: Ratio = (2-ΔCt mc^2^155-Cu) / (2-ΔCt mc2155)(ΔCt = Ct target-Ct control;). The primers are listed in S1 Table.

### Statistical Method

Statistical analysis was carried out with GraphPad Prism 5.0 software. Significant differences in the data were determined by Student’s t test. P values of <0.05 were considered significant.

## Results

### Selection and Growth of Copper-resistant *M*. *Smegmatis* Strain

**Fig 1:**
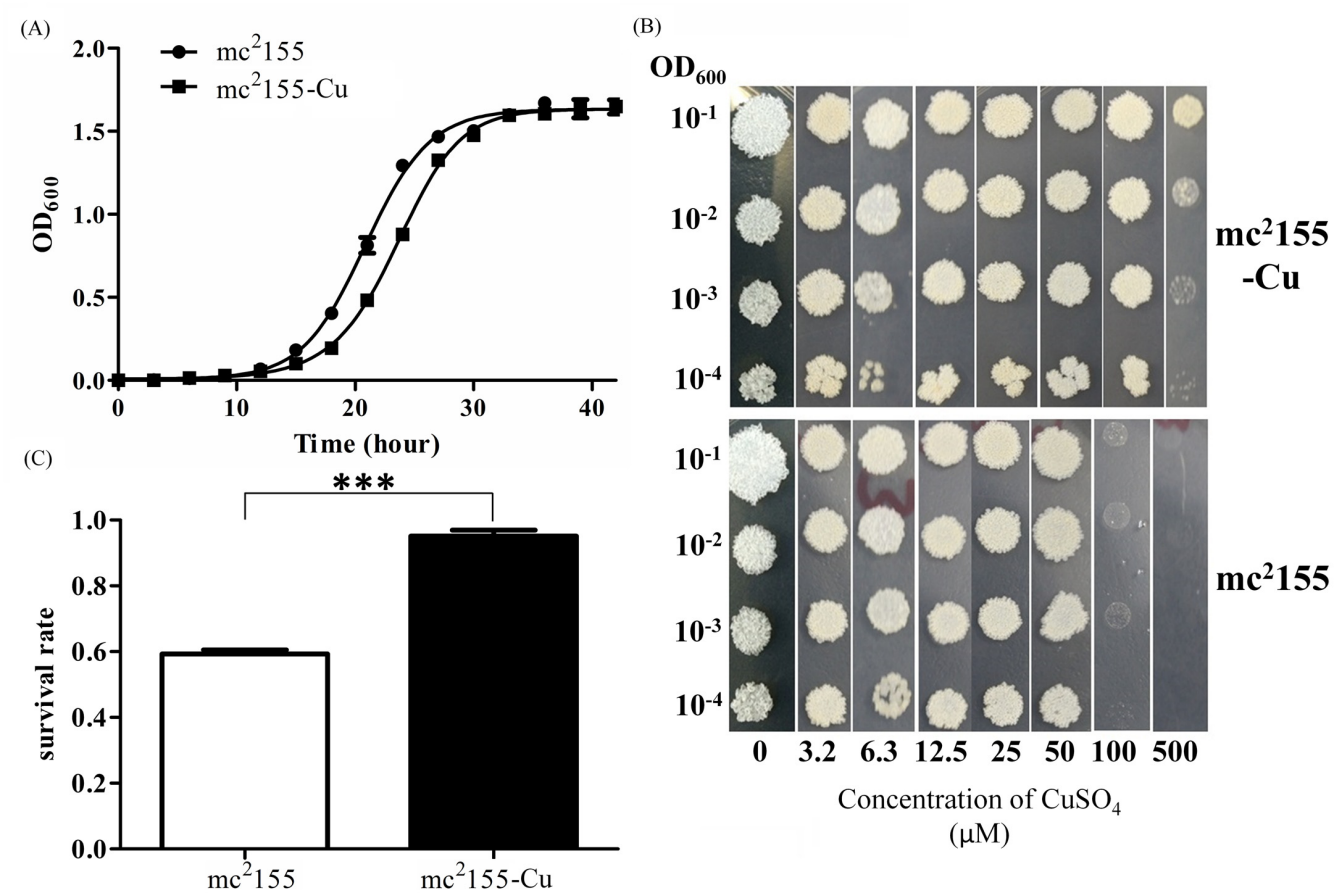
Growth curve and the susceptibility of *M*. *smegmatis* to copper and isoniazid. (a) The growth curve of *M*. *smegmatis* mc^2^155 and the copper resistant strain mc^2^155-Cu were measured in 7H9 media. Experiments were performed in triplicate. Squares, mc2155-Cu strains; circle, mc2155 strain; (b) The bacterial growth on 7H10 plates for *M*. *smegmatis* mc^2^155 and mc^2^155-Cu that were treated with CuSO_4_ at different concentrations for 3 days, respectively. The panels show serial dilution (1:10) of mc^2^155 and mc^2^155-Cu. Diluted M. smegmatis cultures were spotted onto solid 7H10 media in the presence of CuSO_4_ ranged from 0 to 500 μM. Images were taken after 3 days incubation at 37°C. Images stand for 3 independent experiments.; and (c) The bacterial survival rate for *M*. *smegmatis* mc^2^155 and mc^2^155-Cu that were treated with 0.1 mg/ml isoniazid.***p<0.001; n = 3.

Prior to the selection of CuSO_4_–resistant strain, the MIC to CuSO_4_ was determined to be about100 μM in wild type mc^2^155. To establish a CuSO_4_ resistant strain, mc^2^155 was first treated with 50 μM CuSO_4_ and grew into the log phase. Then, bacteria were diluted and treated by gradually increasing CuSO_4_ concentrations. After several rounds of selection, the final concentration of CuSO_4_ reached to 300 μM. To ensure that CuSO_4_-resistant phenotype was stable, bacteria were sub-cultured for 10 generations without CuSO_4_ and then streaked on plates to obtain single colonies. A colony that is highly CuSO_4_ resistant was selected and named mc^2^155-Cu. The growth rates of mc^2^155 and mc^2^155-Cu were monitored at OD_600_ after initial inoculation. As shown in Fig 1(a), the wild type strain mc^2^155 started to grow and entered into lag phase after 16 hr post-inoculation, and then reached the late logarithms/stationary phase after 28 hr post-inoculation. In contrast, mc^2^155-Cu began to grow entered into lag phase after 18 hr and reached the late logarithms/stationary phase after 30 hr post-inoculation. The two hour delay suggested that mc^2^155-Cu grows slower than mc^2^155. The growth of mc^2^155 and mc^2^155-Cu in the presence of different concentrations of CuSO_4_ was displayed in Fig 1(b), showing that mc^2^155-Cu was able to grow in 500 μM CuSO_4_ while mc^2^155 only grew when the concentration of CuSO_4_ was below 100 μM. Based on these results, the MIC of mc^2^155-Cu to CuSO_4_ was estimated to be 500 μM, while that of mc^2^155 was 100 μM. Similarly, MICs of mc^2^155 and mc^2^155-Cu to INH were measured (Table 1), showing that mc^2^155-Cu was more resistant to INH than mc^2^155, but both strains have the similar MICs to Rifampicin. To further confirm that mc^2^155-Cu was relatively more resistant to INH, we performed INH killing assays and found that the survival rate of mc^2^155-Cu was statistically higher than that of mc^2^155 in the presence of 0.1 mg/ml INH (Fig 1(c)).

**Table 1:** The MIC of copper, isoniazid and rifampicin in *M*. *smegmatis* mc^2^155 and the copper resistant strain.

Strain	Copper	Isoniazide	Rifampicin
mc^2^155	100 μM	15 ug/ml	10 ug/ml
mc^2^155-Cu	500 μM	25 ug/ml	10 ug/ml

### Quantitative Proteomic Analysis of mc^2^155-Cu and mc^2^155

**Fig 2:**
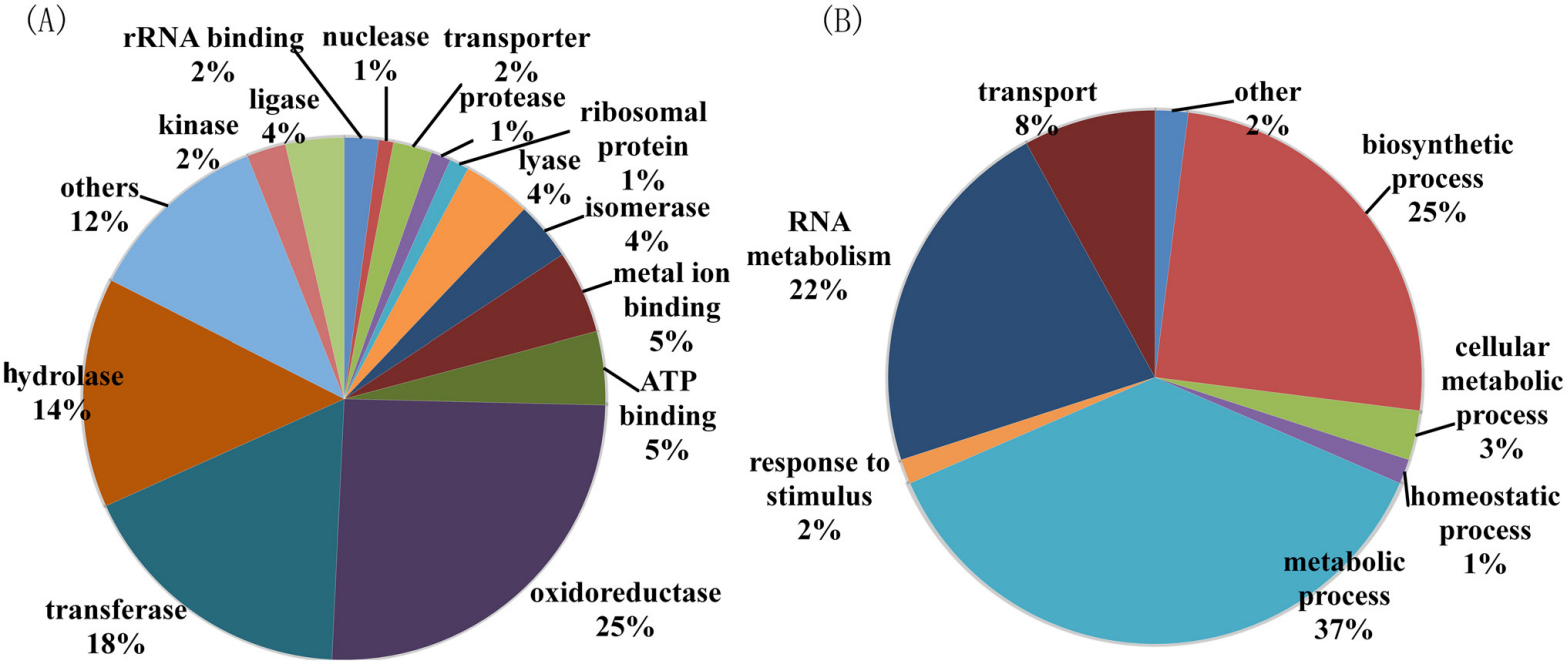
Gene ontology analysis of 345 differentially expressed proteins between *M*. *smegmatis* mc^2^155 and the copper resistant strain. (A) Classification based on protein functions; (B) classification based on the proteins-associated biological processes.

To further characterize changes in bacterial proteome, proteomic analysis was used to find differentially expressed proteins between mc^2^155 and mc^2^155-Cu. An equal amount of proteins from mc^2^155 and mc^2^155-Cu cells were in-solution digested and labeled with TMT reagents. The generated tryptic peptides were fractionated using off-line HPLC and each fraction were further analyzed by nano-LC-MS/MS. Differentially expressed proteins were identified and quantified using TMT-based quantitation. We identified 2799 proteins in two repeated experiments and the false-positive rate was estimated to be less than 1%. Based on reporter ion ratios (>1.5 or <0.7), 345 proteins were found to be differentially expressed between mc^2^155 and mc^2^155-Cu, in which 283 proteins were down-regulated and 62 were up-regulated (S2 and S3 Tables). In order to understand the biological relevance of the identified proteins, the Gene Ontology (GO) was used to cluster the differentially expressed proteins according to their molecular functions and biological processes. The annotations of gene lists are summarized via a pie plot based on the functional classification from Uniprot as shown in Fig 2(a). About 25% of all differentially expressed proteins are oxidoreductases, 18% are transferases, and 14% are hydrolases, indicating that copper ions induce a significant change in cellular redox processes. Three hundred and forty five proteins participated in a variety of cellular processes including metabolic process, biosynthetic process, RNA metabolism, and transport (Fig 2(b)). It is worth mentioning that 15 out of 16 transporter proteins are downregulated and copper-translocating P-type ATPase is the only protein upregulated. All differentially expressed proteins are also classified based on KEGG pathway analysis, indicating that 231 proteins are associated with metabolism and half of those are enzymes in carbohydrate metabolism and amino acid metabolism (S1 Fig). We also noticed that glutamate associated proteins were down-regulated in mc^2^155-Cu including Glutamate binding protein, Glutamate dehydrogenase, Glutamate-ammonia-ligase adenylyltransferase, Glutamate—tRNA ligase, Glutamine synthetase 1, and Glutamyl-tRNA reductase.

### Verification of Differentially Expressed Proteins by qPCR

**Fig 3:**
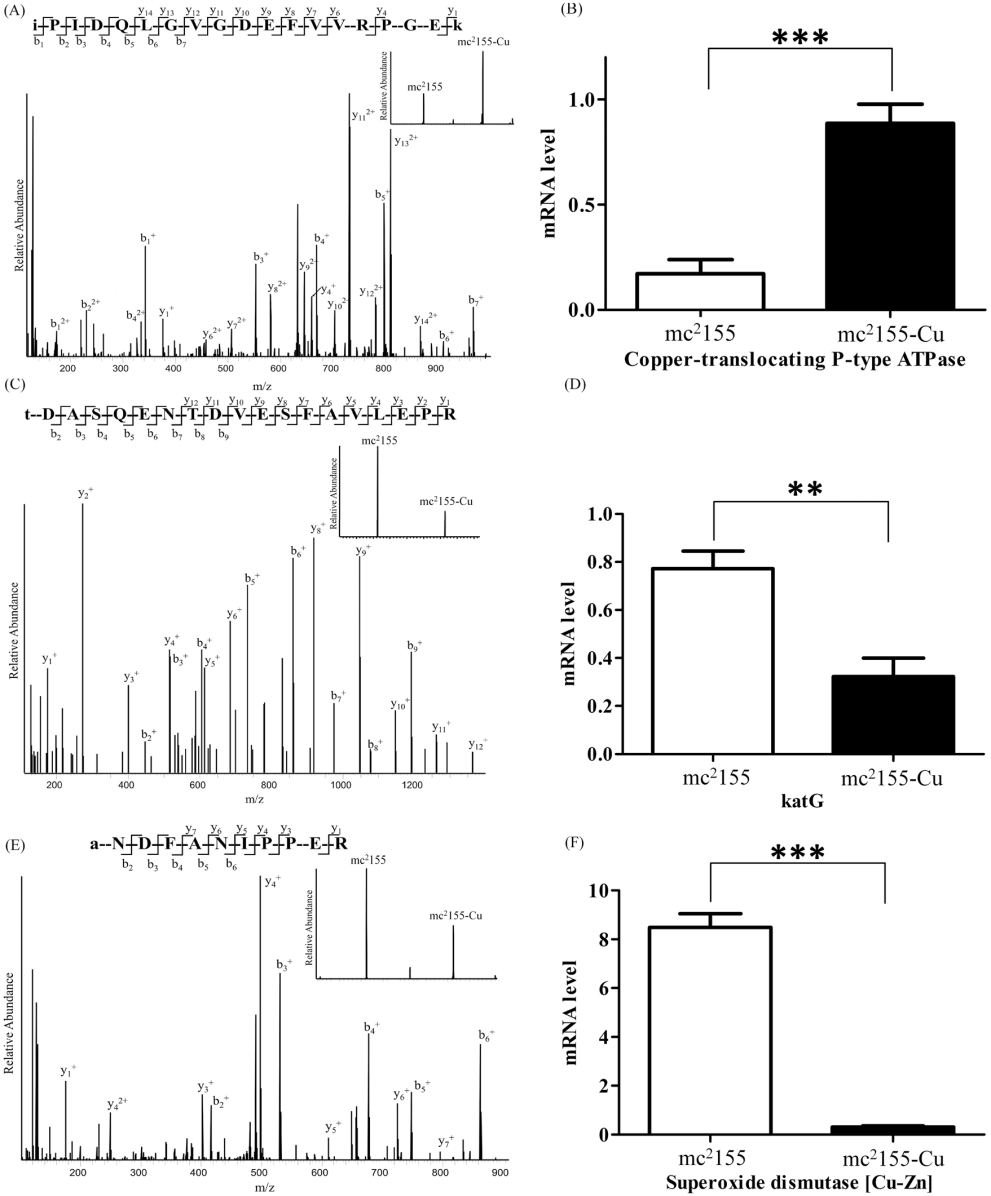
Confirmation of differentially expressed proteins in mc^2^155 and mc^2^155-Cu by quantitative proteomics and qPCR analysis of mRNA expression of copper-translocating P-type ATPase, katG and superoxide dismutase [Cu-Zn]. (a) The MS/MS spectrum of a TMT-labeled peptide from copper-translocating P-type ATPase. The peptide sequence is iPIDQLGVGDEFVVRPGEk. The insert shows intensities of the reported ions at *m/z*130.141 (mc^2^155) and *m/z* 131.138 (mc^2^155-Cu); (b) the mRNA expression of copper-translocating P-type ATPase; (c) The MS/MS spectrum of a TMT-labeled peptide from katG2. The peptide sequence is tDASQENTDVESFAVLEPR. The insert shows intensities of the reported ions at *m/z*130.141 (mc^2^155) and *m/z* 131.138 (mc^2^155-Cu); (d) the mRNA expression of katG2; (e) The MS/MS spectrum of a TMT-labeled peptide from superoxide dismutase [Cu-Zn]. The peptide sequence is aNDFANIPPER. The insert shows intensities of the reported ions at *m/z*130.141 (mc^2^155) and *m/z*131.138 (mc^2^155-Cu); (f) the mRNA expression of superoxide dismutase [Cu-Zn]. **p<0.01; ***p<0.001; n = 3.

Among the differentially expressed proteins between mc^2^155 and mc^2^155-Cu strains, copper-translocating P-type ATPase, Catalase-peroxidase 2 (katG2) and superoxide dismutase [Cu-Zn] are directly associated with copper and INH resistance and cellular redox state. To confirm the results of quantitative proteomic analysis, we first quantified the change of mRNA of these three genes (Fig 3). Quantitative proteomics showed that KatG2 was down-regulated in mc^2^155-Cu (Fig 3(c)) and its mRNA level was also down-regulated by qPCR analysis (Fig 3(d)). The mRNA expressions for copper-translocating P-type ATPase and superoxide dismutase [Cu-Zn] were up-regulated in mc^2^155-Cu, which were consistent with changes in protein expressions (Fig 3(a) and 3(b)). As shown in Fig 3(b), the mRNA expression of copper-translocating P-type ATPase in mc^2^155-Cu is about 4-fold higher than that in mc^2^155, while the ratio of the protein expression is about 3 in mc^2^155-Cu (Fig 3(b)) as compared to mc^2^155. We also carried out qPCR analysis on other proteins including ABC transporter permease/ATP-binding protein, LprG protein, Immunogenic protein MPB64/MPT64, PorinMspA, Proline-rich 28 kDa antigen, Pup-protein ligase, Rv1174c, Transcriptional repressor, CopY family protein and Ribosome binding factor A (S2 Fig). The mRNA levels of all these proteins except Ribosome binding factor A are down-regulated in mc^2^155-Cu strain, in consistent with the quantitative proteomic results.

### Identification of CuSO_4_ mediated changes in cell metabolism

**Fig 4:**
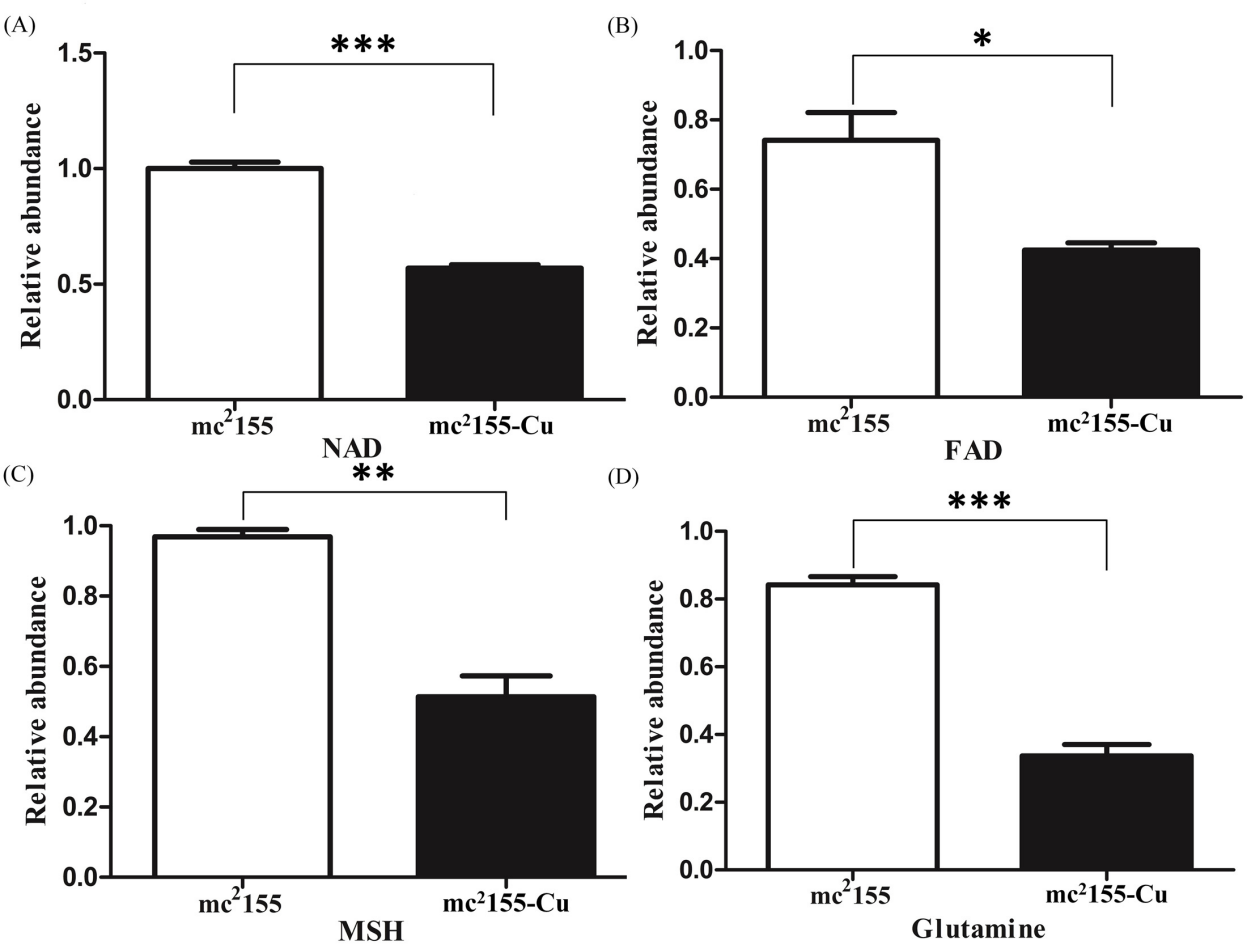
Intracellular concentrations of NAD, FAD, MSH, and glutamine levels in mc^2^155 and mc^2^155-Cu. Relative levels of NAD, FAD, mycothiol (MSH) and glutamine were determined by LC-MS/MS analysis. (a) NAD, (b) FAD, (c) mycothiol (MSH) and (d) glutamine. *p<0.05; **p<0.01; ***p<0.001;n = 3.

Furthermore, we compared the difference in metabolites between mc^2^155-Cu and mc^2^155. Metabolites from mycobacteria were extracted with a cold mixing solvent that contained H_2_O, acetonitrile, and methanol (2:4:4). Using LC-MS/MS analysis, relative levels of NAD, FAD, mycothiol (MSH) and glutamine were determined, showing concentrations of these metabolites were lower in mc^2^155-Cu than those in mc^2^155 (Fig 4). The level of glutamine in mc^2^155-Cu is a third of that in mc^2^155, while levels of other three metabolites were decreased by half in mc^2^155-Cu.

## Discussion

Copper ions acting as a co-factor for enzymes in electron transfer reactions are essential for the survival of organisms from bacteria to mammals while excess copper ions are toxic to cells. Studies showed that host cells used copper ions as a component of the immune system to eliminate intracellular bacteria. As the most successful intracellular pathogen, *M*. *tuberculosis* has evolved many strategies to survive and persist in phagosomes of macrophages, including detoxification strategies to scavenge copper ions [14–21]. To understand effects of copper ions on proteome and metabolome of mycobacteria, we established a copper-resistant strain of *M*. *smegmatis* in the present study, which had a 5-fold higher MIC of copper ions than the wild type mc^2^155 does (Table 1). The copper-resistant strain grew slower and exhibited the higher resistance to INH as compared to mc^2^155 (Fig 1).

Quantitative proteomics showed that 345 proteins out of 2799 identified proteins were differentially expressed between mc^2^155 and mc^2^155-Cu, in which 283 proteins were down-regulated. By GO analysis, it was found that the most differentially expressed proteins were oxidoreductases, suggesting copper ions mainly induced changes in cellular redox processes. NAD and FAD are cofactors of oxidoreductases including key metabolic enzymes such as glyceraldehyde 3-phosphate dehydrogenase and pyruvate dehydrogenase [22]. Decreases in NAD and FAD levels in mc^2^155-Cu can down-regulate activities of glycolytic enzymes, resulting in a decrease in growth rate as compared to mc^2^155. Out of 345 differentially expressed proteins, 231 proteins are associated with cell metabolism and 114 proteins are enzymes in amino acid metabolism and carbohydrate metabolism. Most proteins in amino acid biosynthesis and carbohydrate metabolism are down-regulated, suggesting the copper-resistant bacteria are metabolic inactive as compared to mc^2^155 bacteria.

Previous studies showed that the up-regulation of CptV gene was a major factor to export copper ions from the cell. In this study, both protein and mRNA expressions of copper-translocating P-type ATPase was upregulated in mc^2^155-Cu, while the other ABC transporters including ABC Fe^3+^-siderophores transporter, permease/ATP-binding protein, and ABC CydDC cysteine exporter were down-regulated. The copper-translocating P-type ATPase of *M*. *smegmatis* has a high sequence similarity to CptV and CptA genes in *M*. *tuberculosis*. In *M*. *tuberculosis*, treatment by excess copper ions induced up-regulation of CptV and other transporters including permease and sulfate transporter [23], suggesting that different mycobacterial species may have different responses to copper ion-induced stress, but up-regulation of copper-translocating P-type ATPase is a general mechanism responsible for copper resistance in mycobacteria and other bacterial species [22, 24–26].

**Fig 5:**
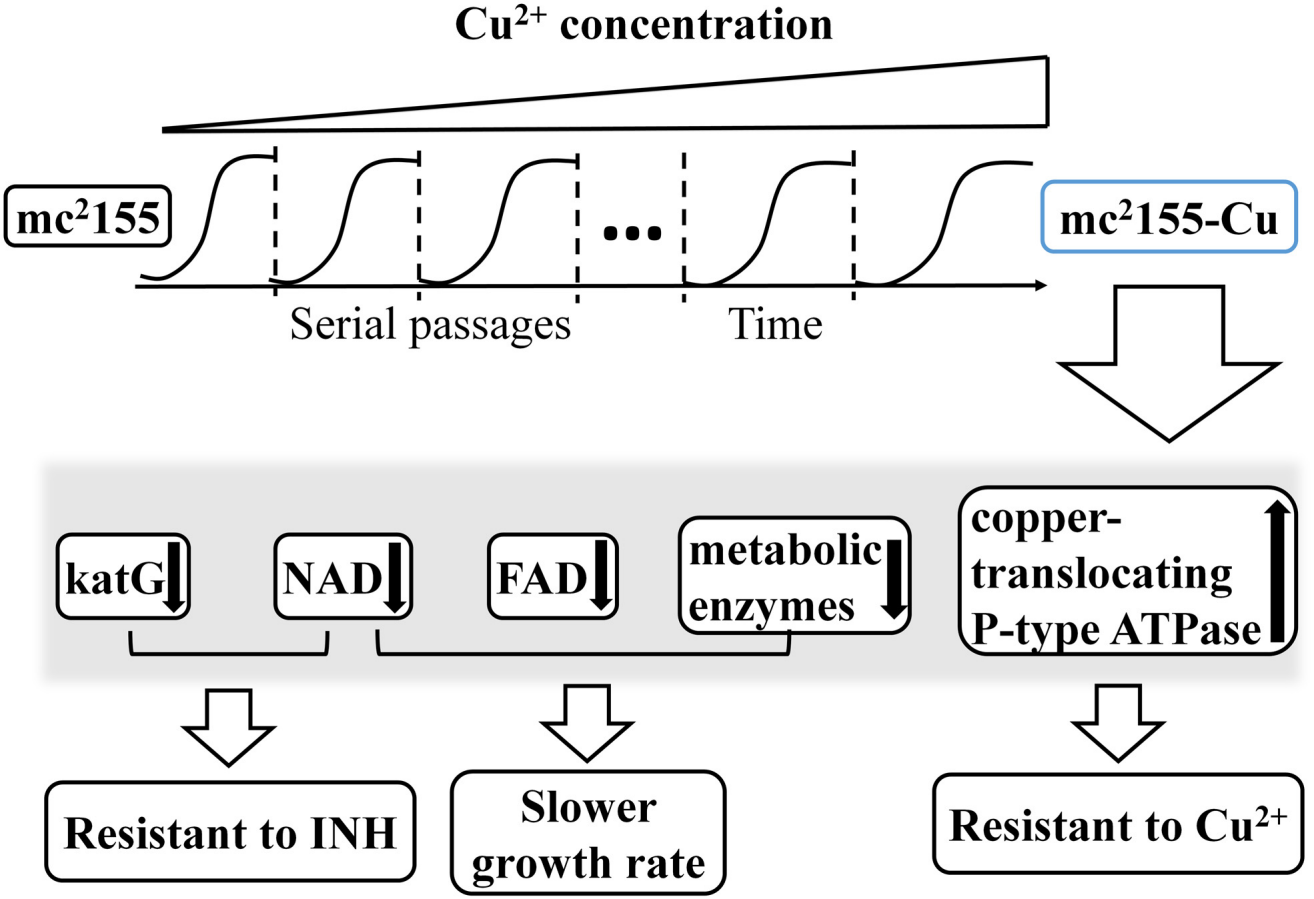
Schematic representation of co-evolution of Copper and INH Resistance in *M*. *smegmatis*. Copper treatment induces the up-regulation of copper translocating P-type ATPase for enhancing bacterial resistance to copper and the down regulation of katG and NAD for increasing the bacterial resistance to INH.

In accompanying to copper resistance, mc^2^155-Cu also acquired the higher resistance to INH than mc^2^155 strain as determined by bacterial growth rates and survival rates in the presence of 0.1 mg/ml INH (Fig 1). INH is the first-line medication in prevention and treatment of TB [27]. As a prodrug, INH needs to be activated by KatG to execute its antibiotic function. KatG is a bifunctional enzyme with both catalase and peroxidase activity and catalyzes the coupling of INH with NAD^+^ to form the isonicotinic acyl-NAD complex, which binds to the enoyl-acyl carrier protein reductase to inhibit the synthesis of mycolic acid required for the mycobacterial cell wall. In the present study, quantitative proteomic analysis showed that the expression level of KatG was down-regulated in mc^2^155-Cu as compared to mc^2^155 (S3 Table and Fig 3). Down-regulation of KatG expression as well as a decrease in cellular NAD level results in the higher resistance to INH in mc^2^155-Cu. On the other hand, no difference in resistance to rifampicin was found between mc^2^155-Cu and mc^2^155. Rifampicin inhibits bacterial DNA-dependent RNA polymerase to prevent RNA synthesis [28] and is not associated with the cellular redox processes. Taken together, our results suggest that co-evolution of copper ion and INH resistance is oxidative stress driven process, as schematically represented in Fig 5. *M*. *tuberculosis* lives and replicates in the host cells. To adopt the hostile environment, intracellular *M*. *tuberculosis* changes its proteome in order to resist stresses including copper ions. Our results suggest that the long lived *M*. *tuberculosis* is resistant to INH and the copper resistant *M*. *smegmatis* is a useful model for finding an effective drug candidate to treat dormant tuberculosis.

## Conclusions

In summary, we developed a copper resistant strain of *M*. *Smegmatis* mc^2^155-Cu, which has a 5 fold higher resistance to copper ions than mc^2^155. Decreases in NAD and glutamine levels and down-regulation of oxidoreductases in mc^2^155-Cu contribute to its slow growth rate as compared to mc^2^155. In consistent with the earlier reports, up-regulation of copper-translocating P-type ATPase in mc^2^155-Cu was observed by quantitative proteomics and qPCR, suggesting that this protein plays a crucial role in copper resistance by exporting intracellular copper ions out. More importantly, the down-regulation of KatG was identified in proteomics that contributes to the high resistance to INH in mc^2^155-Cu strain, indicating the co-evolution of copper and INH resistance. Results presented herein are useful resources to further our understanding of the multifactorial mechanisms of copper resistance in mycobacteria.

## Supporting Information

S1 Fig KEGG pathway analysis of 345 differentially expressed proteins between *M*. *smegmatis* mc^2^155 and the copper resistant strain. (TIFF)

S2 Fig Confirmation of differentially expressed proteins in mc^2^155 and mc^2^155-Cu by qPCR analysis of mRNA expression of (a) ABC transporter permease/ATP-binding protein, (b) LprG protein, (c) Immunogenic protein MPB64/MPT64, (d) PorinMspA, (e) Proline-rich 28 kDa antigen, (f) Pup-protein ligase, (g) Rv1174c, (h) Transcriptional repressor, CopY family protein and (i) Ribosome binding factor A. **p< 0.01, ***p< 0.001; n = 3.
(TIF)

S1 Table Primers used for RT-PCR analysis in this work. (DOCX)

S2 Table Down-regulated proteins in mc^2^155-Cu as compared to mc^2^155. (DOCX)

S3 Table Up-regulated proteins in mc^2^155-Cu as compared to mc^2^155. (DOCX)
