# Supplementary figures and images for: Proteomic Analysis of Drug-Resistant Mycobacteria: Co-Evolution of Copper and INH Resistance
Source: PLoS One. 2015 Jun 2;10(6):e0127788. doi: 10.1371/journal.pone.0127788 (PMC4452738; doi:10.1371/journal.pone.0127788)

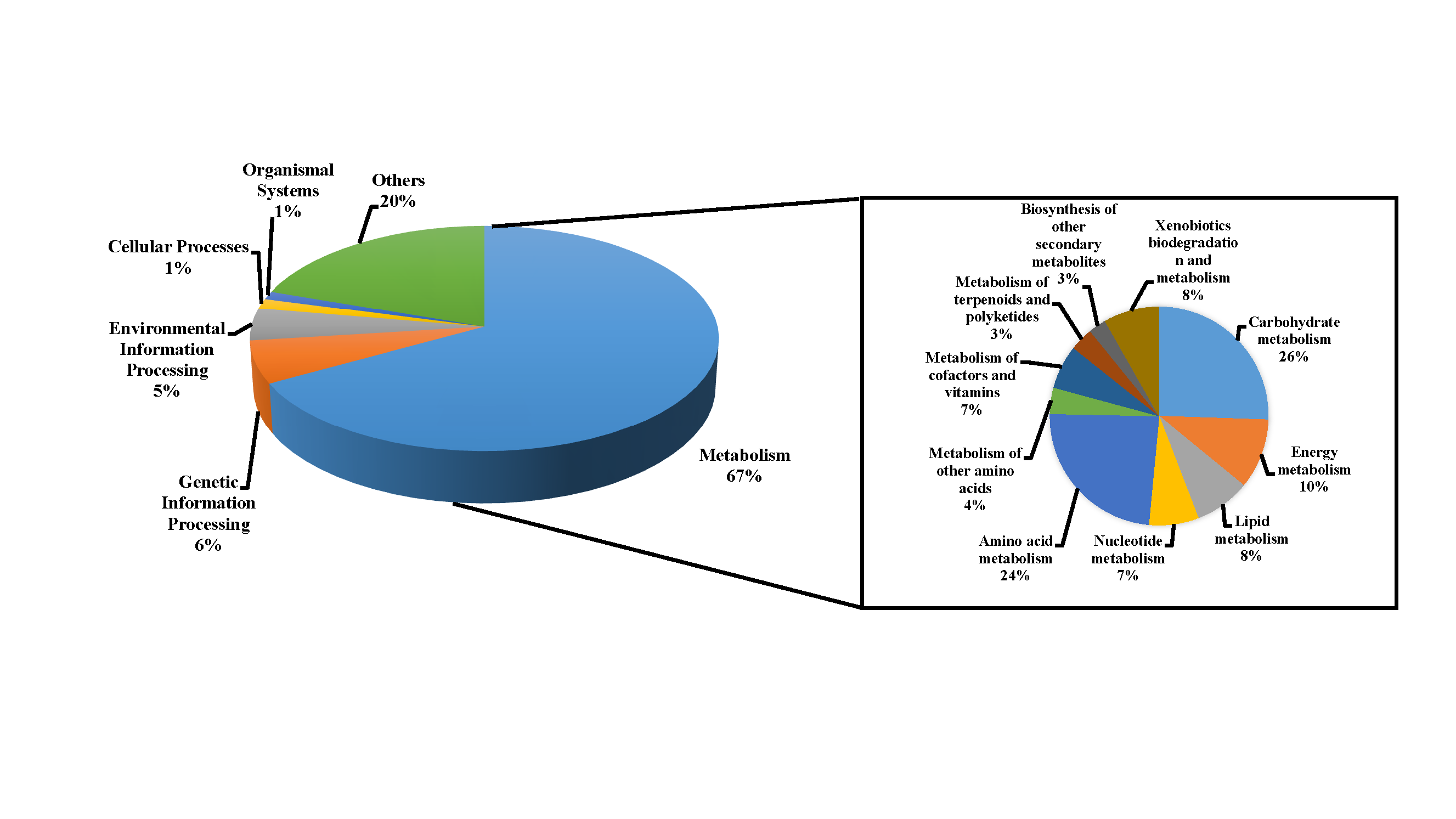

Supplement: S1 Fig — (TIFF) [file pone.0127788.s001.tiff]
