# Supplementary material for: Proteomic Analysis of Drug-Resistant Mycobacteria: Co-Evolution of Copper and INH Resistance
Source: PLoS One. 2015 Jun 2;10(6):e0127788. doi: 10.1371/journal.pone.0127788 (PMC4452738; doi:10.1371/journal.pone.0127788)
Supplement: S1 Table — (DOCX) [file pone.0127788.s003.docx]

S1 table. Primers used in this work

| Primer name | Primer sequence |
| --- | --- |
| rpoD-F | TCTTCGGCGTTGAGC |
| rpoD-R | GTCCGGCGACTTCGT |
| Copper-translocating P-type ATPase CtpA-F | TCGGATTCTGGCTCGGCACAGG |
| Copper-translocating P-type ATPase CtpA-R | CGCGGCCCGTACCGACCAG |
| katG-F | CGGCGAGGAAGACGAATGGC |
| katG-R | CGGCGAGCGGGTCGGGTT |
| Superoxide dismutase [Cu-Zn]-F | GACGGCTCGGGCAAGCTGGTG |
| Superoxide dismutase [Cu-Zn]-R | CGCCGTTGACCTGCTGGTAGCG |
| Transcriptional repressor, CopY family protein-F | GACGAACTGGTCGCCGGGCTCA |
| Transcriptional repressor, CopY family protein-R | CGGCAGAACCCGCTGCTTGCT |
| Proline-rich 28 kDa antigen-F  Proline-rich 28 kDa antigen-R  Rv1174c-F | CCGACCGCACCAGCAAGGACC  CGGTGGTACGCCAGCCGAGAAG  GCACTGCGGTCATCGCCTGGTC |
| Rv1174c-R | CGGCGTCGCGGTGGGTGC |
| Porin MspA-F | CTCGGCTACCAGATCGGCTTCC |
| Porin MspA-R | GAACGGCGGAGCGGTGATGT |
| Pup--protein ligase-F | CCCGCCCGCACGATCAGA |
| Pup--protein ligase-R | CGCGGAATGCGACAACCTGATA |
| ABC transporter, permease/ATP-binding protein-F | GCCTGCGGCGGCTGTTCG |
| ABC transporter, permease/ATP-binding protein-R | GGGTGACCTCGCCGTTGAAGAA |
| LprG protein-F | CCGTGGCGTCGGAGAAGTTGG |
| LprG protein-R | CTGATCGCGTTCGGCCAGAAGA |
| Immunogenic protein MPB64/MPT64-F | CAACGCGACCTACCGGCTGGAT |
| Immunogenic protein MPB64/MPT64-R | CGGGCGTCCAGCACATACGG |
| Ribosome-binding factor A RbfA-F | AGGACCGCGAGCTGCTGGAAT |
| Ribosome-binding factor A RbfA-R | CGTTGGCCTTGCGGATCGTGT |
