# Supplementary material for: Proteomic Analysis of Drug-Resistant Mycobacteria: Co-Evolution of Copper and INH Resistance
Source: PLoS One. 2015 Jun 2;10(6):e0127788. doi: 10.1371/journal.pone.0127788 (PMC4452738; doi:10.1371/journal.pone.0127788)
Supplement: S2 Table — (DOCX) [file pone.0127788.s004.docx]

S2 table. The list of down-regulated proteins in in copper resistant M. smegmatis compared to *M. smegmatis* mc^2^155

| Accession | Description | Score | Ratio |
| --- | --- | --- | --- |
| A0QV29 | [Protein-PII] uridylyltransferase | 35.4 | 0.6 |
| A0R710 | 1-aminocyclopropane-1-carboxylate deaminase | 17.5 | 0.6 |
| A0R5F8 | 2-dehydro-3-deoxy-6-phosphogalactonate aldolase | 13.2 | 0.6 |
| A0QWD3 | 2-hydroxy-6-oxo-6-phenylhexa-2,4-dienoate hydrolase | 13.0 | 0.7 |
| A0R5Q2 | 2-isopropylmalate synthase | 38.6 | 0.7 |
| A0R2P0 | 2-Nitropropane dioxygenase | 12.1 | 0.7 |
| A0QSL6 | 30S ribosomal protein S11 | 17.1 | 0.6 |
| A0QVQ3 | 30S ribosomal protein S15 | 18.2 | 0.4 |
| A0QSE0 | 30S ribosomal protein S17 | 63.3 | 0.7 |
| A0QUZ0 | 3-isopropylmalate dehydratase small subunit | 67.1 | 0.5 |
| A0R4R3 | 3-ketosteroid-9-alpha-hydroxylase oxygenase subunit | 19.4 | 0.5 |
| A0QVD5 | 3-oxoacyl-[acyl-carrier-protein] reductase | 10.6 | 0.7 |
| A0R4S9 | 3-oxosteroid 1-dehydrogenase | 22.7 | 0.7 |
| A0QYI8 | 4-alpha-glucanotransferase | 16.4 | 0.6 |
| A0QQ04 | 4-aminobutyrate transaminase | 12.5 | 0.4 |
| A0R3D7 | 4-diphosphocytidyl-2-C-methyl-D-erythritol kinase | 13.3 | 0.6 |
| A0QSD3 | 50S ribosomal protein L23 | 25.0 | 0.7 |
| A0R6Q9 | 5-methyltetrahydropteroyltriglutamate--homocysteine methyltransferase | 104.7 | 0.7 |
| A0QY21 | 6-aminohexanoate-cyclic-dimer hydrolase | 26.6 | 0.7 |
| A0QWQ1 | AAA ATPase, central region | 10.7 | 0.6 |
| A0R0Z4 | ABC Fe3+-siderophores transporter, periplasmic binding protein | 18.4 | 0.5 |
| A0QWL0 | ABC transporter ATP-binding protein | 32.9 | 0.7 |
| A0R771 | ABC transporter ATP-binding protein | 16.5 | 0.8 |
| A0R1I1 | ABC transporter binding protein | 14.0 | 0.5 |
| A0QS64 | ABC transporter, ATP-binding protein | 29.2 | 0.5 |
| A0QXA6 | ABC transporter, CydDC cysteine exporter (CydDC-E) family protein, permease/ATP-binding protein CydD | 28.0 | 0.6 |
| A0QYH0 | ABC transporter, permease/ATP-binding protein | 19.2 | 0.4 |
| A0R1C3 | ABC-transporter protein, ATP binding component | 100.5 | 0.7 |
| A0QRS0 | ABC-type transport system periplasmic substrate-binding protein | 38.0 | 0.5 |
| A0R364 | Acetamidase/Formamidase family protein | 32.7 | 0.7 |
| A0QUX7 | Acetolactate synthase small subunit | 30.2 | 0.6 |
| A0R4Z5 | Acetyl-CoA acetyltransferase | 42.7 | 0.7 |
| A0QYT0 | Acetylglutamate kinase | 26.1 | 0.5 |
| A0R2V6 | Acyl-[ACP] desaturase | 71.3 | 0.5 |
| A0R6D7 | Acyl-CoA dehydrogenase domain protein | 16.4 | 0.7 |
| A0QQD3 | Acyl-CoA dehydrogenase | 13.1 | 0.7 |
| A0QUH4 | Acyl-CoA dehydrogenase | 18.8 | 0.6 |
| A0QZR2 | Acyl-CoA dehydrogenase | 14.2 | 0.7 |
| A0R1C7 | Acyltransferase family protein | 11.1 | 0.5 |
| A0QR12 | Acyltransferase | 10.6 | 0.7 |
| A0QTL0 | Acyltransferase, ws/dgat/mgat subfamily protein | 13.6 | 0.5 |
| A0R091 | Adenylate cyclase, putative | 15.6 | 0.6 |
| A0R4I6 | Adenylosuccinate lyase | 41.9 | 0.7 |
| A0QQH7 | Adenylosuccinate synthetase | 66.5 | 0.6 |
| A0QYD3 | Alanine and proline-rich secreted protein apa | 59.2 | 0.7 |
| A0QP86 | Aldehyde dehydrogenase family protein | 15.0 | 0.7 |
| A0QUC9 | Aldehyde dehydrogenase | 19.2 | 0.6 |
| A0QVJ6 | Aldehyde dehydrogenase | 20.9 | 0.7 |
| A0R0A7 | Alkyl hydroperoxide reductase/ Thiol specific antioxidant/ Mal allergen | 15.7 | 0.5 |
| A0R2P2 | Alpha-methylacyl-CoA racemase, putative | 18.7 | 0.6 |
| A0QQ51 | Amidohydrolase family protein | 21.2 | 0.6 |
| A0QVC5 | Amine oxidase | 104.5 | 0.7 |
| A0QNQ9 | AMP-binding enzyme, putative | 13.1 | 0.7 |
| A0QU51 | Antigen 85-C | 21.4 | 0.6 |
| A0R624 | Antigen 85-C | 14.0 | 0.7 |
| A0QQA8 | Aspartate aminotransferase | 13.1 | 0.8 |
| A0R201 | ATP synthase gamma chain | 54.2 | 0.7 |
| A0R203 | ATP synthase subunit b-delta | 127.9 | 0.7 |
| A0R200 | ATP synthase subunit beta | 165.2 | 0.7 |
| A0QNI9 | ATPase, AAA family protein | 45.3 | 0.6 |
| A0R196 | ATP-dependent Clp protease ATP-binding subunit ClpX | 57.9 | 0.7 |
| A0R198 | ATP-dependent Clp protease proteolytic subunit 2 | 25.4 | 0.7 |
| A0R6L2 | ATP-dependent helicase HrpA | 25.4 | 0.6 |
| A0R2C3 | Bacterial extracellular solute-binding protein | 52.1 | 0.6 |
| A0QWJ4 | Bacterial extracellular solute-binding protein, family protein 5 | 14.3 | 0.6 |
| A0R261 | Bacterial extracellular solute-binding protein, family protein 5 | 22.2 | 0.7 |
| A0QVY3 | Biotin sulfoxide reductase | 11.0 | 0.7 |
| A0R522 | Biphenyl-2,3-diol 1,2-dioxygenase | 20.5 | 0.7 |
| A0QXC0 | Branched-chain amino acid ABC transporter substrate-binding protein | 104.8 | 0.6 |
| A0R2V8 | CalR9 protein | 55.9 | 0.4 |
| A0QUJ1 | Carnitinyl-CoA dehydratase | 10.4 | 0.7 |
| A0QSA5 | Carveol dehydrogenase | 13.1 | 0.5 |
| A0QXX7 | Catalase-peroxidase 2 | 119.3 | 0.5 |
| A0QZ77 | Cation-transporting ATPase Pma1 | 10.2 | 0.7 |
| A0QYF2 | CBS domain protein | 10.5 | 0.7 |
| A0R0S9 | CBS domain protein | 12.1 | 0.6 |
| A0R7K1 | Chromosomal replication initiator protein DnaA | 10.1 | 0.4 |
| A0R417 | Citrate synthase | 121.3 | 0.6 |
| A0QVL5 | Cob(I)alamin adenosyltransferase | 12.8 | 0.7 |
| Q3L893 | Conserved domain protein | 12.6 | 0.7 |
| A0R4P2 | Conserved domain protein | 29.8 | 0.7 |
| A0R452 | Cupin domain protein | 18.1 | 0.6 |
| A0QNQ7 | Cyclase/dehydrase family protein | 12.6 | 0.5 |
| A0R040 | Cyclase/dehydrase | 21.4 | 0.7 |
| A0R481 | Cyclase/dehydrase | 15.5 | 0.8 |
| A0QS49 | Cyclopropane-fatty-acyl-phospholipid synthase 1 | 36.7 | 0.5 |
| A0R5R5 | Cyclopropane-fatty-acyl-phospholipid synthase | 108.8 | 0.5 |
| Q9L8R4 | CydC | 14.2 | 0.6 |
| A0R5Y9 | Cysteine desulfurase family protein | 11.3 | 0.4 |
| A0R057 | Cytochrome c oxidase subunit 2 | 55.3 | 0.7 |
| A0QT21 | Cytosine/purine/uracil/thiamine/allantoin permease family protein | 12.4 | 0.5 |
| A0QWF4 | Dehydrogenase/reductase SDR family protein member 1 | 13.7 | 0.7 |
| A0R3S3 | D-mannonate oxidoreductase | 10.9 | 0.6 |
| A0QPN2 | DNA gyrase subunit B-like protein MSMEG_0457 | 29.7 | 0.7 |
| A0R564 | DNA integrity scanning protein DisA | 17.4 | 0.5 |
| A0QX55 | DNA polymerase III alpha subunit | 25.3 | 0.5 |
| A0R692 | DNA protection during starvation protein | 11.5 | 0.7 |
| A0R4L1 | DNA-binding response regulator PhoP | 27.3 | 0.7 |
| A0R177 | D-oliose 4-ketoreductase | 12.1 | 0.6 |
| A0QTF9 | dTDP-RhA:a-D-GlcNAc-diphosphoryl polyprenol, a-3-L-rhamnosyl transferase | 11.6 | 0.7 |
| A0R376 | Ectoine/hydroxyectoine ABC transporter, ATP-binding protein | 14.1 | 0.5 |
| A0R0Y9 | Elongation factor 4 | 32.5 | 0.7 |
| A0QWR4 | Elongation factor P | 23.0 | 0.5 |
| A0QT19 | Endoribonuclease L-PSP superfamily protein | 36.1 | 0.6 |
| A0QT74 | Enoyl-CoA hydratase/isomerase family protein | 12.5 | 0.7 |
| A0QX16 | Enoyl-CoA hydratase/isomerase | 10.9 | 0.6 |
| A0R088 | Enoyl-CoA hydratase/isomerase | 33.8 | 0.7 |
| A0QQW5 | Eptc-inducible aldehyde dehydrogenase | 55.0 | 0.7 |
| A0QYW4 | Excinuclease ABC, A subunit | 55.9 | 0.6 |
| A0R5R4 | FAD binding domain protein | 40.1 | 0.7 |
| A0R665 | FAD-containing monooxygenase EthA | 21.0 | 0.5 |
| A0QT77 | Fatty acid desaturase | 60.0 | 0.6 |
| A0QTL4 | Fatty acid desaturase | 16.3 | 0.6 |
| P00215 | Ferredoxin | 62.9 | 0.7 |
| A0QX00 | FeS assembly protein SufD | 40.3 | 0.7 |
| A0QNG7 | FHA domain protein | 72.1 | 0.6 |
| A0QT20 | Flavin-containing monooxygenase FMO | 48.5 | 0.7 |
| A0QPG8 | Fmt protein | 17.6 | 0.8 |
| A0R2I4 | FO synthase | 49.0 | 0.7 |
| A0QXS0 | Formamidase | 10.3 | 0.7 |
| A0R0F4 | Formamidase | 27.1 | 0.7 |
| A0R342 | Formamidase | 47.7 | 0.6 |
| A0QNJ2 | Ftsk/spoiiie family protein | 16.5 | 0.7 |
| A0QVJ4 | Gamma-glutamylisopropylamide synthetase | 11.1 | 0.7 |
| O68956 | Glucosamine--fructose-6-phosphate aminotransferase [isomerizing] | 32.8 | 0.6 |
| A0QP90 | Glucose-6-phosphate 1-dehydrogenase | 67.0 | 0.6 |
| A0QPS6 | Glucosidase | 32.5 | 0.6 |
| A0QVX3 | Glutamate binding protein | 55.3 | 0.7 |
| A0R3E3 | Glutamate dehydrogenase | 22.8 | 0.7 |
| A0R082 | Glutamate-ammonia-ligase adenylyltransferase | 11.5 | 0.7 |
| A0QUY7 | Glutamate--tRNA ligase | 85.0 | 0.6 |
| A0R079 | Glutamine synthetase 1 | 311.9 | 0.4 |
| A0R5P1 | Glutamine synthetase, type III | 22.1 | 0.7 |
| A0R5T7 | Glutamine-binding periplasmic protein/glutamine transport system permease protein | 19.0 | 0.5 |
| A0QR17 | Glutamyl-tRNA reductase | 19.6 | 0.5 |
| A0R729 | Glycerol kinase 3 | 299.5 | 0.7 |
| A0R727 | Glycerol operon regulatory protein | 16.2 | 0.7 |
| A0R1C6 | Glycerol-3-phosphate acyltransferase | 25.5 | 0.6 |
| A0QT70 | Glycerol-3-phosphate dehydrogenase 2 | 33.1 | 0.4 |
| A0R6D2 | Glycogen debranching enzyme GlgX | 63.3 | 0.6 |
| A0R2E2 | Glycogen synthase | 18.4 | 0.7 |
| A0QUV4 | Glycosyl hydrolase, family protein 57 | 10.5 | 0.7 |
| A0R425 | Glyoxalase family protein | 32.8 | 0.7 |
| A0QR82 | Glyoxalase/bleomycin resistance protein/dioxygenase | 19.7 | 0.6 |
| A0QNU4 | GntR-family protein transcriptional regulator | 29.6 | 0.5 |
| A0QXY7 | GTP cyclohydrolase | 23.0 | 0.7 |
| A0QSZ0 | Homoserine O-acetyltransferase | 20.8 | 0.8 |
| A0QUN5 | Hydrogenase accessory protein HypB | 33.6 | 0.5 |
| A0QUN9 | Hydrogenase expression/formation protein HypD | 24.5 | 0.5 |
| A0QUM7 | Hydrogenase-2, large subunit | 89.8 | 0.6 |
| A0QUM6 | Hydrogenase-2, small subunit | 35.6 | 0.4 |
| A0QYW5 | Hydrolase | 26.3 | 0.7 |
| A0R7G6 | Inositol-3-phosphate synthase | 154.0 | 0.5 |
| P41403-2 | Isoform Beta of Aspartokinase | 63.0 | 0.7 |
| A0QX46 | Isoleucine--tRNA ligase | 65.8 | 0.6 |
| A0R548 | Lipoprotein | 12.0 | 0.7 |
| A0R2Q5 | Long-chain specific acyl-CoA dehydrogenase | 35.3 | 0.5 |
| A0R562 | LpqE protein | 12.9 | 0.5 |
| A0QWU8 | LprG protein | 20.0 | 0.7 |
| A0QXV6 | L-serine ammonia-lyase | 17.1 | 0.7 |
| A0QYS4 | Macrolide-transport ATP-binding protein abc transporter | 26.4 | 0.6 |
| A0R0B3 | Meromycolate extension acyl carrier protein | 49.4 | 0.7 |
| A0QVT2 | Metallo-beta-lactamase superfamily protein | 20.0 | 0.5 |
| A0QVP6 | Metallophosphoesterase | 25.5 | 0.7 |
| A0QQX8 | Methoxy mycolic acid synthase 1 | 99.5 | 0.5 |
| A0QSJ2 | Methylmalonate-semialdehyde dehydrogenase | 45.4 | 0.6 |
| A0QX36 | Methylmalonyl-CoA mutase large subunit | 90.3 | 0.7 |
| A0QNZ7 | Monoglyceride lipase | 41.4 | 0.6 |
| A0QYQ6 | MutT/nudix family protein | 21.9 | 0.7 |
| A0QTX2 | N5,N10-methylenetetrahydromethanopterin reductase-related protein | 28.2 | 0.7 |
| A0QNN8 | NAD(P) transhydrogenase, alpha subunit | 33.9 | 0.6 |
| A0R2N3 | NAD-dependent deacetylase | 12.6 | 0.7 |
| A0QSY4 | NADH:flavin oxidoreductase/nadh oxidase | 10.2 | 0.5 |
| A0QW43 | NADPH-dependent fmn reductase | 11.6 | 0.6 |
| A0QUN3 | NHL repeat protein | 11.5 | 0.6 |
| A0QP15 | O-acetylhomoserine/O-acetylserine sulfhydrylase | 21.5 | 0.7 |
| A0QYH8 | Oxidoreductase | 21.2 | 0.7 |
| A0R1A9 | Oxidoreductase | 26.7 | 0.6 |
| A0QZF6 | Oxidoreductase, 2OG-Fe(II) oxygenase family protein | 15.0 | 0.7 |
| A0QUM8 | Peptidase M52, hydrogen uptake protein | 10.7 | 0.8 |
| A0QWK5 | Peptidyl-prolyl cis-trans isomerase, cyclophilin-type | 29.2 | 0.7 |
| A0QNF2 | Periplasmic binding protein | 21.9 | 0.7 |
| A0QR99 | Periplasmic binding protein | 30.9 | 0.7 |
| A0QQS6 | Phosphatidylserine decarboxylase proenzyme | 13.9 | 0.6 |
| A0QWX4 | Phosphoenolpyruvate carboxylase | 95.8 | 0.7 |
| A0QUA6 | Phosphoglucomutase, alpha-D-glucose phosphate-specific | 84.9 | 0.7 |
| A0QP89 | Phosphogluconate dehydratase | 80.3 | 0.6 |
| A0QRB9 | Phosphohydrolase | 19.2 | 0.6 |
| A0QRA0 | Phosphoserine phosphatase | 31.5 | 0.6 |
| A0R524 | Pigment production hydroxylase | 34.0 | 0.7 |
| A0QQY0 | Polyphosphate kinase 2 superfamily protein | 26.8 | 0.7 |
| A0QR04 | Ppx/GppA phosphatase family protein | 20.6 | 0.7 |
| A0R613 | Probable arabinosyltransferase B | 13.7 | 0.6 |
| A0R5E1 | Probable cold shock protein A | 69.9 | 0.4 |
| A0QP12 | Probable conserved membrane protein | 11.1 | 0.7 |
| A0R582 | Probable conserved transmembrane protein rich in alanine | 11.9 | 0.5 |
| A0R426 | Probable ferredoxin/ferredoxin--NADP reductase | 11.5 | 0.5 |
| A0R083 | Probable glutamine synthetase 2 | 77.7 | 0.7 |
| A0QPS9 | Probable sugar ABC transporter, substrate-binding protein, putative | 11.8 | 0.7 |
| A0R708 | Probable transcriptional regulator YdhC | 11.4 | 0.6 |
| A0QR90 | Probable transcriptional regulatory protein | 16.5 | 0.4 |
| A0QQW4 | Prolyl oligopeptidase family protein | 31.2 | 0.6 |
| A0QTE7 | Propionyl-CoA carboxylase beta chain | 147.9 | 0.7 |
| A0QZ47 | Proteasome subunit beta | 32.2 | 0.7 |
| A0QQC9 | Protein grpE | 33.6 | 0.7 |
| A0QZ42 | Pup--protein ligase | 48.7 | 0.6 |
| A0R0Z5 | Puromycin N-acetyltransferase | 12.9 | 0.5 |
| A0R465 | Putative 3-hydroxyacyl-CoA dehydrogenase | 94.3 | 0.7 |
| A0QPV4 | Putative acyl-CoA dehydrogenase | 85.0 | 0.8 |
| A0QYR7 | Putative Clp protease subunit | 52.6 | 0.5 |
| A0QUZ6 | Putative cystathionine gamma-synthase | 13.9 | 0.5 |
| A0R6A3 | Putative cytochrome P450 135B1 | 13.6 | 0.6 |
| Q9RP37 | Putative glycine cleavage system H protein GcvH (Fragment) | 22.8 | 0.4 |
| A0R5P5 | Putative oxidoreductase | 19.6 | 0.7 |
| A0R175 | Putative oxidoreductase YisS | 23.2 | 0.5 |
| A0R518 | Putative short-chain type dehydrogenase/reductase MSMEG_6031 | 13.1 | 0.7 |
| A0R4Q0 | Putative succinate-semialdehyde dehydrogenase [NADP(+)] | 22.5 | 0.6 |
| A0QNH8 | Pyridoxamine 5'-phosphate oxidase family protein | 11.2 | 0.7 |
| A0R0B0 | Pyruvate dehydrogenase E1 component | 166.8 | 0.5 |
| A0R171 | Pyruvate synthase | 99.9 | 0.5 |
| A0R5U3 | Queuine tRNA-ribosyltransferase | 13.7 | 0.7 |
| A0QYX6 | Regulatory protein GntR, HTH | 14.5 | 0.7 |
| A0QPW1 | Regulatory protein, MarR | 11.5 | 0.6 |
| P0CG99 | Ribonucleoside-diphosphate reductase subunit alpha 1 | 92.5 | 0.7 |
| A0QR94 | Ribonucleoside-diphosphate reductase, beta subunit | 24.2 | 0.5 |
| A0R3D9 | Ribosomal RNA small subunit methyltransferase A | 58.3 | 0.7 |
| Q2YHI1 | Rmt4 | 55.7 | 0.5 |
| A0R408 | Sensor-type histidine kinase PrrB | 12.4 | 0.6 |
| A0R3L2 | Serine/threonine-protein kinase PknE | 31.3 | 0.7 |
| A0R4M3 | Short chain dehydrogenase | 22.6 | 0.6 |
| A0QVZ1 | Soluble pyridine nucleotide transhydrogenase | 15.5 | 0.7 |
| A0QXF4 | Spermidine/putrescine ABC transporter ATP-binding subunit | 10.5 | 0.7 |
| A0R2W6 | Steroid delta-isomerase | 16.3 | 0.6 |
| A0R183 | Sugar ABC transporter substrate-binding protein | 17.6 | 0.7 |
| A0QTE3 | Sulfurtransferase | 74.1 | 0.5 |
| A0QXB3 | Sulfurtransferase | 23.0 | 0.6 |
| A0QQQ1 | Superoxide dismutase [Cu-Zn] | 31.9 | 0.6 |
| A0QUN1 | Tetratricopeptide repeat domain protein | 43.4 | 0.7 |
| A0R1Y5 | Tetratricopeptide repeat domain protein | 29.5 | 0.7 |
| A0R2F0 | Tetratricopeptide repeat family protein | 20.0 | 0.6 |
| A0QTR3 | TetR-family protein transcriptional regulator | 25.0 | 0.7 |
| A0R315 | TetR-family protein transcriptional regulator | 17.0 | 0.8 |
| A0R346 | TetR-family protein transcriptional regulator | 11.1 | 0.7 |
| A0QQH8 | Thioesterase family protein | 14.2 | 0.7 |
| Q3L894 | TnpR | 16.1 | 0.7 |
| A0QTG7 | TobH protein | 12.9 | 0.6 |
| A0R089 | Transcription regulator AmtR | 17.1 | 0.7 |
| A0QZ09 | Transcriptional regulator, CadC | 10.5 | 0.7 |
| A0R561 | Transcriptional regulator, CarD family protein | 34.5 | 0.6 |
| A0R5H1 | Transcriptional regulator, Crp/Fnr family protein | 89.1 | 0.6 |
| A0QZJ0 | Transcriptional regulator, GntR family protein | 27.3 | 0.6 |
| A0QXK4 | Transcriptional regulator, IclR family protein, putative | 18.6 | 0.7 |
| A0QYF9 | Transcriptional regulator, MerR family protein | 15.5 | 0.7 |
| A0QUD0 | Transcriptional regulator, putative | 14.8 | 0.7 |
| A0QR73 | Transcriptional regulator, TetR family protein | 19.6 | 0.6 |
| A0QUI9 | Transcriptional regulator, TetR family protein | 24.3 | 0.4 |
| A0R1R5 | Transcriptional regulator, TetR family protein | 24.9 | 0.7 |
| A0QUZ9 | Transcriptional regulatory protein, AsnC family protein | 11.7 | 0.5 |
| A0QSL3 | Translation initiation factor IF-1 | 16.6 | 0.5 |
| A0QS43 | Translocase | 32.1 | 0.7 |
| A0R0W9 | Trehalase | 62.4 | 0.7 |
| A0R4M9 | Trehalose-phosphate synthase | 22.3 | 0.6 |
| A0QZ58 | tRNA (Adenine-N(1)-)-methyltransferase | 10.3 | 0.4 |
| A0R7I2 | tRNA adenylyltransferase | 27.5 | 0.6 |
| A0R4B5 | tRNA-dihydrouridine synthase | 24.3 | 0.7 |
| A0QP21 | Two component response transcriptional regulatory protein prra | 13.4 | 0.7 |
| A0QSK9 | Two-component sensor histidine kinase | 19.2 | 0.7 |
| A0QRU1 | Type III restriction enzyme, res subunit | 31.5 | 0.6 |
| A0R052 | Ubiquinol-cytochrome c reductase cytochrome b subunit | 22.3 | 0.5 |
| A0R629 | UDP-galactopyranose mutase | 36.1 | 0.7 |
| A0R6Q0 | Uncharacterized protein MSMEG_6630 | 34.4 | 0.8 |
| A0R0S4 | Undecaprenyl diphosphate synthase | 15.8 | 0.5 |
| A0QRZ8 | Uricase | 24.9 | 0.5 |
| A0QRN3 | Urocanate hydratase | 12.1 | 0.6 |
| A0QVL7 | Uroporphyrin-III C-methyltransferase | 26.4 | 0.4 |
| A0QQG1 | Xanthine dehydrogenase | 13.8 | 0.7 |
| A0QRZ5 | Xanthine/uracil permeases family protein | 15.7 | 0.5 |
| A0QUG7 | ZbpA protein | 22.0 | 0.7 |
