# Supplementary material for: Proteomic Analysis of Drug-Resistant Mycobacteria: Co-Evolution of Copper and INH Resistance
Source: PLoS One. 2015 Jun 2;10(6):e0127788. doi: 10.1371/journal.pone.0127788 (PMC4452738; doi:10.1371/journal.pone.0127788)
Supplement: S3 Table — (DOCX) [file pone.0127788.s005.docx]

S3 Table. The list of 65 up-regulated proteins in copper resistant M. smegmatis compared to *M. smegmatis* mc^2^155

| Accession | Description | Score | Ratio |
| --- | --- | --- | --- |
| A0R2G5 | 2,3,4,5-tetrahydropyridine-2,6-dicarboxylate  N-succinyltransferase | 67.4 | 1.7 |
| A0R102 | 30S ribosomal protein S20 | 27.8 | 1.8 |
| A0R087 | 3-methyl-2-oxobutanoate hydroxymethyltransferase | 18.2 | 1.6 |
| A0QUH2 | 3-oxoacyl-(Acyl-carrier-protein) reductase | 14.1 | 1.6 |
| A0QUW5 | 4-coumarate:CoA ligase | 16.5 | 1.6 |
| A0R2T2 | 4-hydroxy-3-methylbut-2-enyl diphosphate reductase | 13.6 | 1.5 |
| A0QSL9 | 50S ribosomal protein L17 | 30.3 | 1.8 |
| A0QSG5 | 50S ribosomal protein L18 | 24.8 | 1.8 |
| A0QSG0 | 50S ribosomal protein L24 | 37.6 | 2.9 |
| A0R215 | 50S ribosomal protein L31 | 44.4 | 1.6 |
| A0QSD2 | 50S ribosomal protein L4 | 65.2 | 1.9 |
| P80673 | 60 kDa chaperonin (Fragment) | 39.5 | 28.1 |
| A0QWX6 | 6-phosphogluconolactonase | 50.2 | 1.7 |
| A0QS90 | Alcohol dehydrogenase, class IV | 85.1 | 2.2 |
| A0QVT5 | Antibiotic biosynthesis monooxygenase domain protein | 11.7 | 1.5 |
| A0R1H3 | Antioxidant, AhpC/TSA family protein | 66.0 | 1.7 |
| A0QSY5 | Bifunctional protein FolD | 65.9 | 1.7 |
| A0QTF5 | Biotin-[acetyl-CoA-carboxylase] ligase | 21.9 | 1.7 |
| A0R1G3 | Clavaldehyde dehydrogenase | 17.2 | 1.5 |
| A0QSP0 | Coenzyme B12-dependent glycerol dehydrogenase small subunit | 14.0 | 2.7 |
| A0R277 | Copper-translocating P-type ATPase | 43.8 | 3.1 |
| A0QW08 | Deoxyuridine 5'-triphosphate nucleotidohydrolase | 12.6 | 1.5 |
| Q9ZHC5 | DNA-binding protein HU homolog | 168.6 | 1.7 |
| A0QXC8 | DoxX subfamily protein, putative | 14.9 | 2.3 |
| A0QWT7 | Esterase | 16.6 | 1.6 |
| Q6XXM0 | Fatty acid synthetase I | 627.7 | 2.1 |
| A0QVC8 | Glyoxylate reductase | 27.0 | 1.6 |
| A0QXK5 | Hydrolase | 111.6 | 3.2 |
| A0R576 | Lsr2 protein | 16.1 | 1.7 |
| A0R0Q6 | MarR-family protein transcriptional regulator | 16.9 | 1.8 |
| A0QWN0 | Metallopeptidase, zinc binding | 11.7 | 1.6 |
| A0QW27 | Methionine-R-sulfoxide reductase | 17.1 | 1.6 |
| A0QZ33 | Nitrilase/cyanide hydratase and apolipoprotein N-acyltransferase | 19.0 | 1.6 |
| O85501 | Nucleoside diphosphate kinase | 55.1 | 1.6 |
| A0QXK7 | Oxidoreductase, FAD/FMN-binding | 79.5 | 2.3 |
| A0QNQ5 | Oxidoreductase, zinc-binding dehydrogenase family protein | 42.7 | 1.6 |
| A0QU07 | Oxidoreductase, zinc-binding dehydrogenase family protein | 52.0 | 1.6 |
| A0QYI2 | Para-nitrobenzyl esterase | 20.1 | 1.6 |
| A0QYB3 | Periplasmic sugar-binding proteins | 17.6 | 2.0 |
| A0R0W2 | Phosphoadenosine phosphosulfate reductase | 12.4 | 1.6 |
| A0R110 | Phosphoglycerate mutase family protein | 17.8 | 1.6 |
| A0R4H2 | Phosphoribosylformylglycinamidine synthase, PurS protein | 14.9 | 1.6 |
| A0QRA7 | Probable enoyl-CoA hydratase | 32.8 | 1.6 |
| A0QZ48 | Prokaryotic ubiquitin-like protein Pup | 80.9 | 1.7 |
| A0QX98 | Prolipoprotein diacylglyceryl transferase | 23.0 | 1.5 |
| A0QSP2 | Propanediol utilization: dehydratase, medium subunit | 32.5 | 1.9 |
| A0QRJ0 | Protease HtpX homolog | 24.0 | 1.9 |
| A0R025 | Protein MraZ | 11.1 | 1.8 |
| A0QTY3 | Putative HpcE protein | 10.1 | 1.9 |
| A0QVB6 | Putative oxidoreductase | 10.0 | 1.7 |
| A0QWY3 | Quinone oxidoreductase | 61.2 | 1.6 |
| A0QWU9 | Riboflavin synthase, alpha subunit | 20.3 | 2.6 |
| A0QVM8 | Ribosome-binding factor A | 119.4 | 7.6 |
| A0QVZ5 | RNA polymerase sigma factor | 10.8 | 1.5 |
| A0R278 | Secreted protein | 34.4 | 3.4 |
| A0R4Y7 | Short chain dehydrogenase | 16.6 | 1.6 |
| A0QTY6 | Stress responsive A/B Barrel Domain superfamily protein | 17.1 | 1.7 |
| A0R1X3 | ThiS family protein | 27.3 | 2.5 |
| A0QVY9 | Thymidylate synthase | 30.3 | 1.5 |
| A0QT32 | Uracil phosphoribosyltransferase 1 | 44.4 | 1.6 |
| Q9S426 | WhmD | 10.5 | 1.9 |
| A0QTP1 | YbaK/ebsC protein | 13.5 | 1.7 |
